# Annotations

**Published:** 1899-12-09

**Authors:** 


### Annotations.

### Inciting to Procure Abortion.

To those who have gone to much trouble, and probably to considerable expense, in laying bare the machinations of advertising quacks, and especially of those whose advertisements are probably in many cases merely lures to attract unwary women into the hands of the abortion monger, it must be a matter of great satisfaction to find that the trial of a group of persons for conspiring together, and with other persons unknown, to incite women who should read or become cognisant of pamphlets and circulars published in the name of Madame Frain, to attempt to administer to themselves noxious things with intent to procure a miscarriage, has ended in a conviction. To lawyers the case will be very interesting as giving a definition, a ruling on a point which appears not to have been raised before, in regard to what is and what is not in given circumstances a criminal attempt to do a thing, and how far a person inciting is or is not criminally responsible. The conclusion arrived at by the Judge (Mr. Justice Darling) is that if the woman, believing that she is taking a noxious thing within the meaning of the statute, does with intent to procure abortion take a thing in fact harmless, she is guilty of the attempt to procure abortion within the meaning of the statute. Again, if the person inciting the woman to take the thing himself believed the thing to be a noxious drug capable of procuring abortion, he would be guilty of inciting her to attempt to commit the crime, although, owing to facts being otherwise than he believed, the commission of the crime in the manner proposed was impossible. But a person supplying to a woman—knowing that she will take it in the belief that it is a noxious thing, and in order to procure abortion—a thing to his knowledge not capable of procuring abortion does not incite her to attempt to procure abortion, and does not himself commit a crime. According to this definition a great deal evidently depended upon whether or not the drugs used were as a fact noxious. Mr. Justice Darling, however, in giving this decision added that, even assuming the things given to be harmless and worthless, he saw no reason why an indictment for false pretences should not be preferred and prosecuted. On the verdict of guilty being returned by the jury, Mr. Justice Darling gave a significant caution to the newspapers who put in advertisements of this kind, saying that they had now had a warning, and if they continued to do so they might find themselves in the same position as the defendants in this case. Hence this trial has had a very useful ending.

### Consulting Physicians for South Africa.

A suggestion made by the *Practitioner* is worthy of consideration. If the Government has done well in sending out consulting surgeons to our army in South Africa, why not also consulting physicians? "Very soon," we are told, "our men will feel the fiery breath of the African summer, and dysentery and typhoid will attack them. A still deadlier disease may come; indeed, it is already casting its shadow before, for cases of plague are reported at Delagoa Bay." No slur on the army medical officers is meant by this suggestion, but the *Practitioner* maintains that "too many commanding officers share the opinion of the Commander-in-Chief that sanitation is a 'fad,'" and goes on to say, "In Egypt, and also, I believe, in the Soudan, the prevalence of typhoid among the troops was hushed up by order, the cases being returned as simple continued fever. In these campaigns the strongest remonstrances of medical officers against insanitary abominations that could easily have been prevented were met by the military authorities either with stolid indifference or with a blank *non possumus*. For the protection of the forces in South Africa against the dangers that may arise from official ignorance or apathy, the Government would be well advised if they appointed one or two consulting physicians," who, "in addition to their special knowledge, would be, in a disciplinary sense, independent of the military authorities, and could, therefore, offer advice not merely 'when it is wanted,' as Lord Wolseley graciously allows, but when it is not wanted." The presence of such men with the army "would be at once a support to the medical officers and a check on commanders who may think the care of the health of their solders a 'fad.'" Clearly the *Practitioner* is not in love with the War Office.

